# The Essential Role of High-resolution MRI Fistulogram Imaging with Ultrasound Gel Enema in Diagnosing Complex Rectovaginal Fistulas Post-Chemoradiation for Rectal Cancer: A Case Study Review

**DOI:** 10.2174/0115734056453274260523111606

**Published:** 2026-06-10

**Authors:** Mohammad Aljamal, Mousa Shaheen, Zuhal Y. Hamd, Iyad Awad, Abdullah Alabdullah, Mohammed Alharbi, Lama Almudaimeegh

**Affiliations:** 1 Department of Medical Imaging, Faculty of Allied Medical Sciences, Arab American University, 13 Zababdeh, Jenin P.O. Box 240, Palestine; 2 Department of Medical Imaging, Shahin X-ray and MRI Medical Center, Al-Malek Faisal St., Bab Al-Zawyah, Hebron, Palestine; 3 Department of Radiological Sciences, College of Health and Rehabilitation Sciences, Princess Nourah bint Abdulrahman University, Riyadh 11671, Saudi Arabia; 4 Academy of Family Medicine, Riyadh Second Health Cluster, Riyadh, Saudi Arabia; 5 Department of Medical Imaging, King Abdullah bin Abdul-Aziz University Hospital, Riyadh, Saudi Arabia; 6 Department of Internal Medicine, College of Medicine, Princess Nourah Bint Abdulrahman University, Riyadh, Saudi Arabia

**Keywords:** Ultrasound gel, Rectovaginal fistula, Chemoradiation, High-resolution MRI, Diagnostic imaging, Pelvic MRI

## Abstract

**Background:**

Rectovaginal fistula (RVF) is a rare and severe complication that may follow chemoradiation treatment of rectal cancer. The limitations of traditional imaging modalities are due to the diagnostic difficulty of rectovaginal fistula (RVF) caused by vague symptomatology. However, because pelvic MRI fistulogram offers better contrast in soft-tissue and multi-planar image viewing, which are very important for accurate diagnosis and efficient treatment planning, it has become the ultimate choice for the evaluation of RVF.

**Case Presentation:**

A 36-year-old female who had previously been diagnosed with rectal cancer and had undergone a chemoradiation regimen experienced a clinical response to treatment. Subsequently, she returned to our office with complaints of new-onset pelvic pain. The patient underwent a high-resolution pelvic MRI fistulogram using an ultrasound gel as an enema agent. The imaging revealed a well-defined fistulous tract between the rectum and vagina, with uncertain residual mural thickening, which significantly impacted her clinical course.

**Conclusion:**

This case demonstrates how to use high-resolution ultrasound gel to visualize an undetected rectovaginal fistula and reinforces the importance of this technique in understanding associated disorders and helping the interdisciplinary team make decisions regarding RVF.

## 1 INTRODUCTION

Rectovaginal fistulas (RVF) are abnormal connections between the rectum and vagina through which fecal material passes into the vagina [1]. These fistulas are primarily caused by prolonged, obstructed labor in developing countries [2]. Surgical procedures, tumors, and X-ray (radiation) therapy are the main causes [3]. The incidence of rectovaginal fistula (RVF) following chemoradiation or rectal cancer resection is generally less than 3%.[4]. Its incidence is highest in females with low anterior rectal tumors; as a result, surgical procedures are performed with great care in an effort to reduce the risk of RVF. There are many causes of rectovaginal fistula, including Crohn's disease, inflammatory bowel disease (IBD), sexual abuse, and unsafe abortions [5, 6]. Several adverse effects are associated with rectovaginal fistulas, including fecal incontinence, frequent vaginal infections, and emotional distress [7]. Therefore, proper diagnosis and treatment are critically important.

Rectovaginal fistula is a major complication that has a serious negative effect on a person's quality of life and serves to complicate their recovery process. Although it can be avoided, there is no consensus on the best options for patients or on the technical aspects of treating these conditions effectively [8]. After surgical treatment, a rectovaginal fistula is complex and occurs for many different reasons. There are multiple surgical risks involved in the development of rectovaginal fistula, including surgical damage to the septum, as well as leakage associated with anastomosis following low anterior resection with total mesorectal excision. Moreover, the planes of dissection are usually in front of the vaginal wall in a few instances, especially with low rectal tumors, and this exposes them to accidental trauma. Anastomotic leaks occur in 5-15% of cases and may lead to rectovaginal fistulas due to ongoing inflammation and infection [7, 9]. Moreover, the use of minimally invasive techniques has not eliminated this risk. Indeed, current research has found that robotic and laparoscopic techniques produce comparable rates of fistulas to those of open surgical approaches [10].

Radiation therapy increases the risk of injury to the rectal wall as a result of the immediate effects on tissue and later effects of both direct and indirect radiation on both normal and abnormal tissues. In addition, some of the mechanisms by which radiation therapy causes injury to the rectal wall include progressive tissue ischemia, activation of fibroblasts, and damage to the microvascular endothelium. Furthermore, because the latency period for these alterations to develop is often 3-5 months to several years after treatment, they are likely to be associated with fistula formation. There is also a clear dose-response relationship between the development of fistulas and the total dose received in patients treated with more than 50 Gy [11]. Tumors are common in regions that have been irradiated previously, and radiation-damaged tissue is less capable of resuming normal functions after surgical trauma. Another issue that could occur postoperatively, such as surgical site infection (SSI), is one of the most common and significant complications after colorectal surgery, due to the high bacterial load naturally present in the colon and rectum. It is linked to higher postoperative morbidity, extended hospitalization, high readmission rates, and high healthcare costs [12, 13]. This creates a complex interplay of factors that complicates management and prevention strategies. The effects of each factor vary among patients. Surgical complications tend to manifest right away, within weeks to months following a surgery, and radiation-induced fistulas may manifest several years after the treatment is over [14]. The synergistic effect of radiation and chemotherapy on tissue damage is demonstrated, but accurate risk stratification is difficult because people vary in radiosensitivity [15].

Rectovaginal fistulas (RVF) are a complicated disorder that is difficult to diagnose. Clinical tests are sufficient to misdiagnose RVFs, particularly when the fistulous tracts are small or high [16]. Traditionally used imaging techniques have many limitations: conventional contrast fistulography cannot typically evaluate occult tracts, the success of endoanal ultrasound is highly dependent on the operator and can also be impacted by post-radiation therapy effects, and CT scans do not produce optimal soft-tissue resolution [17]. These shortcomings have made pelvic MRI an integral component of the diagnostic workup, as MRI is especially important for providing high soft-tissue contrast and multidimensional images [5].

MRI plays an important role in the management of rectovaginal fistulas (RVF) [18], as evidenced by numerous articles documenting how MRI findings can impact surgical planning for 52% of RVFs, providing surgeons with evidence to base their decisions on before surgery. In addition, surgical repair success rates for RVFs may improve as surgeons gain knowledge of the extent of RVF disease and are able to make informed surgical decisions before performing the repair [19]. MRI findings related to the vascularity of the tissues involved in the fistula, the status of the anal sphincter, and the locations of associated abscesses are all critical components of MRI-derived treatment decision-making [20].

Moreover, other studies assessed the relevance of high-tech imaging methods, including texture analysis and perfusion imaging, for identifying the potential for healing and selecting the most suitable personalized interventions for patients with RVFs. One previous study summarized protocol enhancements for MRI of RVFs. Ideally, T2-weighted imaging in both sagittal and oblique planes will provide a complete anatomic assessment of the RVFs. Additionally, diffusion-weighted imaging will identify any underlying inflammation associated with the RVFs [5, 21]. Dynamic cine sequences can demonstrate intermittent flow through the fistula, while a contrast agent can help differentiate between disease recurrence and post-treatment changes [22]. Advances in 3D isotropic imaging can help visualize detailed tracts with volumetric assessment, which is essential for determining the surgical approach [23].

Rectal contrast enema mixed-contrast MRI is a MRI contrast technique that has been explored for use in addition to MRI's high-contrast resolution protocols to improve visualization of fistula anatomy and pathology. This results in better definition and improved image quality of the fistula, thereby increasing the clinical accuracy of the image. By providing more detailed information than standard imaging methods, this technique also provides relevant data that may not be interpretable with conventional imaging methodologies. Using a rectal enema increases visibility of surrounding soft tissue and helps enlarge the rectal lumen. This improvement is important for the accurate identification of the characteristics of the fistula tract. A commonly used contrast agent for this purpose is a dilute gadolinium-based contrast agent (GBCA), which is considered the gold standard in MR fistula procedures [24]. In a previous study, direct MR fistulography using a hydrogen peroxide and gadolinium mixture outperformed physical examination and conventional low-resolution MRI in detecting internal openings and fistula tracks [25]. However, various other enema solutions may be used depending on specific clinical conditions or safety concerns. A simple saline solution can serve as a “negative” contrast medium for people with severe renal impairment. Since gadolinium-based contrast agents (GBCA) are limited by the risk of nephrogenic systemic fibrosis (NSF) and their high cost, a viscous ultrasound gel solution has been proposed, as it provides higher T2 signal intensity [26]. This technique allows clinicians to visualize the fistula without using any external contrast material and is relatively inexpensive.

The purpose of this study is to assess the value of a high-resolution MRI of the Rectovaginal Fistula using ultrasound gel enema in a case study. It also discusses the clinical implications of the diagnostic use of high-resolution MRI for rectovaginal fistula, using ultrasound gel as an enema. Knowledge of the effectiveness and indications for the use of this imaging modality will be beneficial to patients as the number of such cases continues to increase.

## 2 CASE PRESENTATION

In October 2023, a 36-year-old woman was scanned using a pelvic MRI due to her complaints of pelvic pain, fatigue, and bright red blood in her feces. The MRI was obtained in the clinical setting, prior to and after administration of IV Gadolinium-based contrast agents, using multiplanar imaging (Siemens Esseca MRI) at 1.5 Tesla. MRI assessment included the distal sigmoid colon and the proximal rectum, with the wall at 4-5 mm in thickness. The MRI findings were indicative of possible inflammatory changes, but were not necessarily diagnostic. The MRI findings did not demonstrate any evidence of local lymphadenopathy, pelvic collection, or perirectal soft tissue infiltration. There was no signal of pelvic ascites or suspected lymph nodes, and the uterus, adnexal organs, and bladder were all normal. The hip and sacroiliac joints were also unharmed.

A colonoscopy was necessary to investigate abnormal imaging, which led to the suspicion of a lesion in the rectum. The colonoscopy showed a rigid, fixed ulcerated lesion that was about 5 cm above the anus. Numerous strategic biopsies were obtained based on an anatomically abnormal tissue pattern and examined histologically using biopsy pathology. The pathology findings showed adenocarcinoma, and a diagnosis of rectal cancer was made based on moderately differentiated adenocarcinoma histopathology. After confirming the biopsy pathology report, the oncologist recommended initiating chemotherapy treatment. The chemotherapy regimen included Xeloda (capecitabine) 3,000 mg with external beam radiotherapy to deliver a total dosage of 50 Gy over a 28-day duration, administered as preoperative chemoradiation. The main objective of this approach was to eliminate micrometastatic disease in the locoregional area and achieve tumor downstaging, thereby reducing the risk of recurrence. A follow-up colonoscopy was done eight weeks after the treatment ended to assess the tumor's reaction. No residual tumor mass or ulcerative focus was visible at the initial site, and endoscopic observation showed complete healing of the mucosa. This finding strongly indicates a complete clinical response to the treatment.

In February 2025, the patient experienced new pelvic pain, which led to a comprehensive pelvic MRI evaluation using the same system. The MRI imaging sequence protocols used in this stage are shown in Table **1**. Multiplanar, multisequence T1- and T2-weighted imaging, before and after intravenous contrast administration, was part of the imaging protocol, as shown in Figs. (**1** and **2**). The radiologist reported minimal wall thickening in the proximal rectum and distal sigmoid, with a maximum thickness of approximately 4–5 mm. This finding could be inflammatory. The fistulous tract between the rectum and uterus was not well defined; therefore, the radiologists chose to perform an MRI fistulogram.

The patient adhered to a particular bowel preparation protocol in order to guarantee the best possible image quality for the MRI fistulogram. This protocol involved taking a Normalax tablet orally to aid in colon cleansing and started with a 6-hour fast before the test. The rectal lumen was significantly expanded by inserting an enema tip approximately 7 cm into the rectum. The ultrasound gel used was sterile aqueous ultrasound gel (without dilution), administered by slow, gentle manual injection at room temperature (slightly warmed) to ensure patient comfort. The gel served as a contrast to better visualize the wall of the rectum. Following the administration of the gel intrarectally, a dedicated multiplanar T1- and T2-weighted sequence was acquired (Table **2**). The radiologist identified that there was heterogeneous and irregular thickening of the wall of the rectoanal tract, with a maximum thickness of 1.3 cm and a longitudinal extent of approximately 4.3 cm. This observation is indicative of unremitting or recurrent malignant disease due to the previously known primary tumor.

A major complication was a fistulous tract measuring about 2-3 mm in diameter. This tract connects the back wall of the vagina and the front wall of the rectum, which is located at a distance of approximately 4-5 cm from the anus. There was clear evidence of ultrasound gel leakage from the rectum into the vagina, a hyperintense fistulous tract extending between the anterior rectal wall and the posterior uterine wall in sagittal view (Fig. **3A**) and axial view (Fig. **3B**). Also, there was an incidental oval lesion on the cervix, which was 2.5 x 2 x 3 cm. This lesion showed hyperintensity on T1 and T2 images with mild enhancement, resulting in moderate cervix widening. The patient was referred to a gynecological consultation to rule out potential pathologies, such as a polyp.

According to diagnostic imaging results, the patient's endorectal advancement flap (ERAF) operation was successful. Endoscopic assessment six months after surgery confirmed that the surgical incision was completely healed and had no signs of healing abnormalities or recurrence of the initial fistula; this is normal after colorectal surgery because infections and leakage are common complications. The level of pain experienced by the patient after the surgery significantly reduced, and her overall quality of life significantly improved.

## 3 DISCUSSION

Colorectal surgery leads to significant morbidity and mortality since the post-operative complications of surgery, such as but not limited to surgical site infection (SSI) and anastomotic leakage, are common in surgery. Modern advances in deep learning may assist in diagnosing and predicting the likelihood of post-operative complications using a novel type of convolutional neural network that leverages intra-operative video data, achieving 99.6% accuracy in predicting colorectal anastomotic leakage [27]. The potential use of Butyrylcholinesterase (BChE) levels as an early indicator of SSIs has been identified. BChE levels are reportedly associated with the probability and severity of infection after colon surgery [28].

The presence of a rectovaginal fistula is a serious complication of rectal cancer treatment that greatly diminishes the patient's physical, psychological, and social quality of life. Factors contributing to the development of a rectovaginal fistula include chemotherapy and radiation therapy, as demonstrated in the present case and supported by the literature. The mechanisms underlying the development of a rectovaginal fistula involve a combination of surgical trauma, anastomotic leakage, progressive ischemia, and tissue fibrosis resulting from radiation therapy [29-31]. Both mechanisms are likely associated with the development of a fistula after chemoradiation therapy in this case because of an undetected anastomotic complication. According to the literature [32], this patient received 50 Gy of radiation therapy and was thus considered high risk, underscoring the importance of treatment-related factors in fistula etiology.

The evidence from the diagnostic process for this patient underscores the great challenges and the essential role of modern imaging in the management of rectovaginal fistulas (RVF). Physical examination alone is often insufficient to diagnose rectovaginal fistulas (RVFs), given the non-specific symptoms that usually accompany small or tortuous tracts. [33]. Indeed, focused pelvic MR imaging alone did not provide a definitive diagnosis for the patient’s pelvic pain. The MR imaging protocol followed the current gold standard of multiplanar T2-weighted sequences, intrarectal contrast with ultrasonic gel at the time of imaging, and post-contrast imaging. By this technique, detailed fistulous anatomy was demonstrated at 4-5 cm from the anus with a diameter of 2-3 mm. Other important information obtained by MR included an incidental cervical lesion and suspected residual or recurrent thickening of the rectal wall, underscoring the technique's exceptional capability to comprehensively evaluate the pelvis [34, 35].

The injected contrast material demonstrated the uterine-rectal fistula on MRI; however, the fistula's detection will depend on the radiologist's experience. On the other hand, high-resolution MRI with ultrasound gel makes diagnosing the fistula unproblematic. This means it can be tested without doubt and, therefore, would significantly enhance efficiency, given that specialists have data on the size and location of fistula sites to make better treatment decisions, thus reducing morbidity for patients and decreasing the risk of late recurrence. Thus, high-resolution MRI fistulography using an ultrasound gel enema seems to be a helpful tool in diagnosing and characterizing complex rectovaginal fistulas, particularly in patients who have undergone chemoradiation therapy for rectal cancer. This technique is especially useful because of its ability to clearly visualize fistulous tracts and surrounding connective tissue. Therefore, MRI fistulography following ultrasound gel enema may serve as an important diagnostic modality in fistula assessment and may further contribute to the development of surgical management algorithms.

The imaging findings altered the clinical course, initially suggesting a complete clinical response to chemoradiation, but later revealing a complicating fistulous tract. Evidence of heterogeneous wall thickening on MR imaging strongly suggests that the fistula is secondary to either recurrent or residual neoplasm. This is extremely important, as a benign fistula due to radiotherapy can be surgically repaired, while a malignant fistula most commonly requires palliative treatment [36]. MRI differentiates such diagnoses by demonstrating features such as ill-defined margins and mass-like wall thickening, which directly guide prognosis and treatment. Research shows that MRI strongly influences the surgeon's approach in most patients with complex fistulae, leading to a change in the initially planned surgery in almost one-third of cases [37]. In addition, this would have helped avoid a futile surgical treatment attempt.

Finally, the ability to detect fistulae on MRI, both pre- and post-contrast, depends on the radiologist's expertise and experience. In this regard, using ultrasound gel and high-resolution MRI would be an excellent step forward in determining the site and size of the fistula and, hence, its likelihood of detection, which would make treatment much more precise and targeted, thus saving most of the time. Most importantly, the patient's symptoms of foul-smelling discharge and fecal incontinence are responsible for anxiety and depression, which might have been avoided by a timely, specific diagnosis and appropriate management leading to faster resolution of symptoms.

High-resolution MR- fistulography and ultrasound gel enema are valuable tools in the diagnosis of rectovaginal fistulas. The limitations of this study are the single-case design, which does not allow generalizing the results, and the lack of control groups or the measurement of diagnostic parameters to validate the results. Since inter-operator reliability has not been studied yet, no technical standardization is possible, and the method should not be used in some anorectal conditions. Thus, it is a hypothesis-generating, operator-dependent, and short-term diagnostic study whose results do not rule out imaging and can be applied to supplement imaging in problematic cases. Future research will need to consider factors such as the patient's anatomy, the location of the lesion, rectal compliance, gel properties, and contraindications, such as intolerance to severe rectal irritation or rectal distension. In addition, the extent of standardization ought to be experimented on a larger scale.

## CONCLUSION

In conclusion, high-resolution magnetic resonance imaging with enema preparation using ultrasound gel is useful for identifying ill-defined rectovaginal fistulas (RVF), particularly after chemoradiation treatment for rectal cancer. Ultrasound gel was effective in identifying the location and tract of the fistula. This type of analysis should continue to be performed through an interdisciplinary, “team” approach, which will help provide optimal patient care through clinical evaluation. Further studies assessing the diagnosis and management of RVF using combination techniques will ultimately lead to a better understanding of RVF and provide better evidence-based practice for RVF compared with other diagnostic methods.

## Figures and Tables

**Fig. (1):**
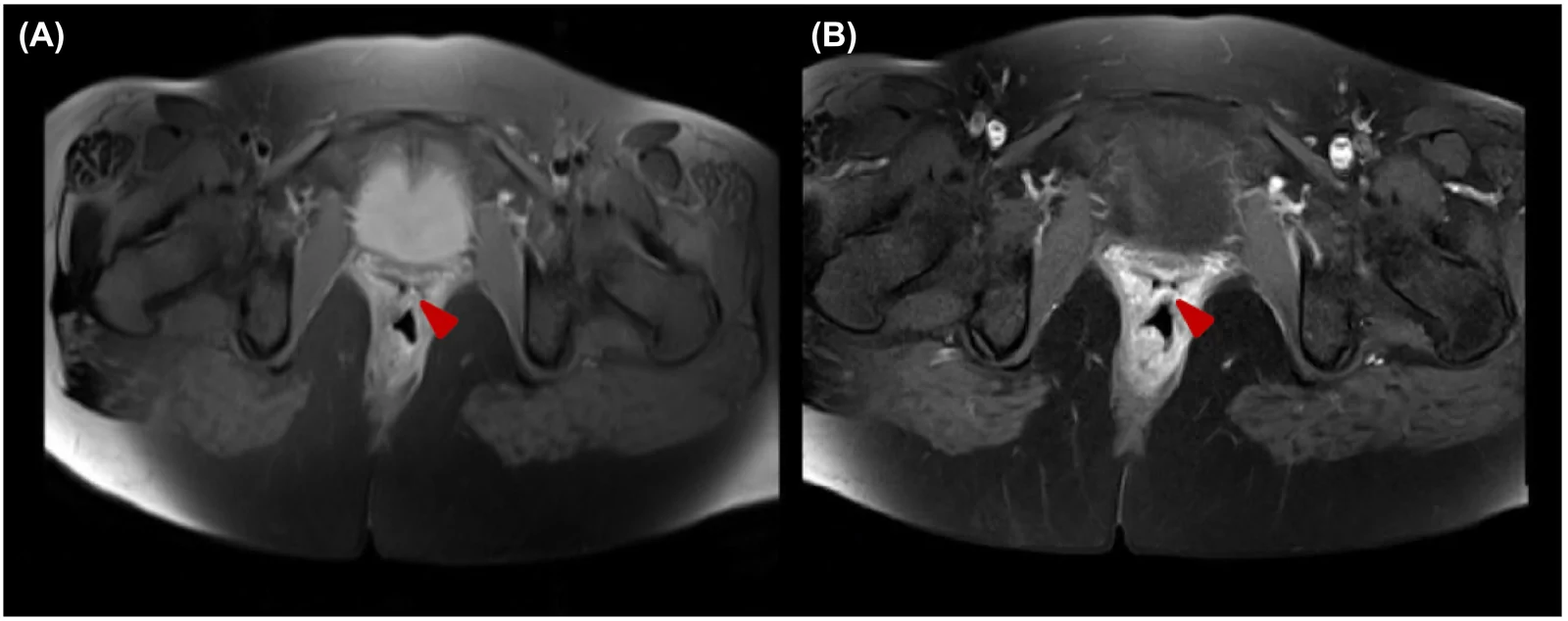
**A**: Axial T2-weighted turbo spin echo (TSE) with fat saturation (FS) pre-contrast acquisition identifies the fistulous tract. **B**: TSE Axis T1-weighted with FS post-contrast imaging shows the fistulous tract between the rectum and uterus (red arrow); however, it was not well defined.

**Fig. (2):**
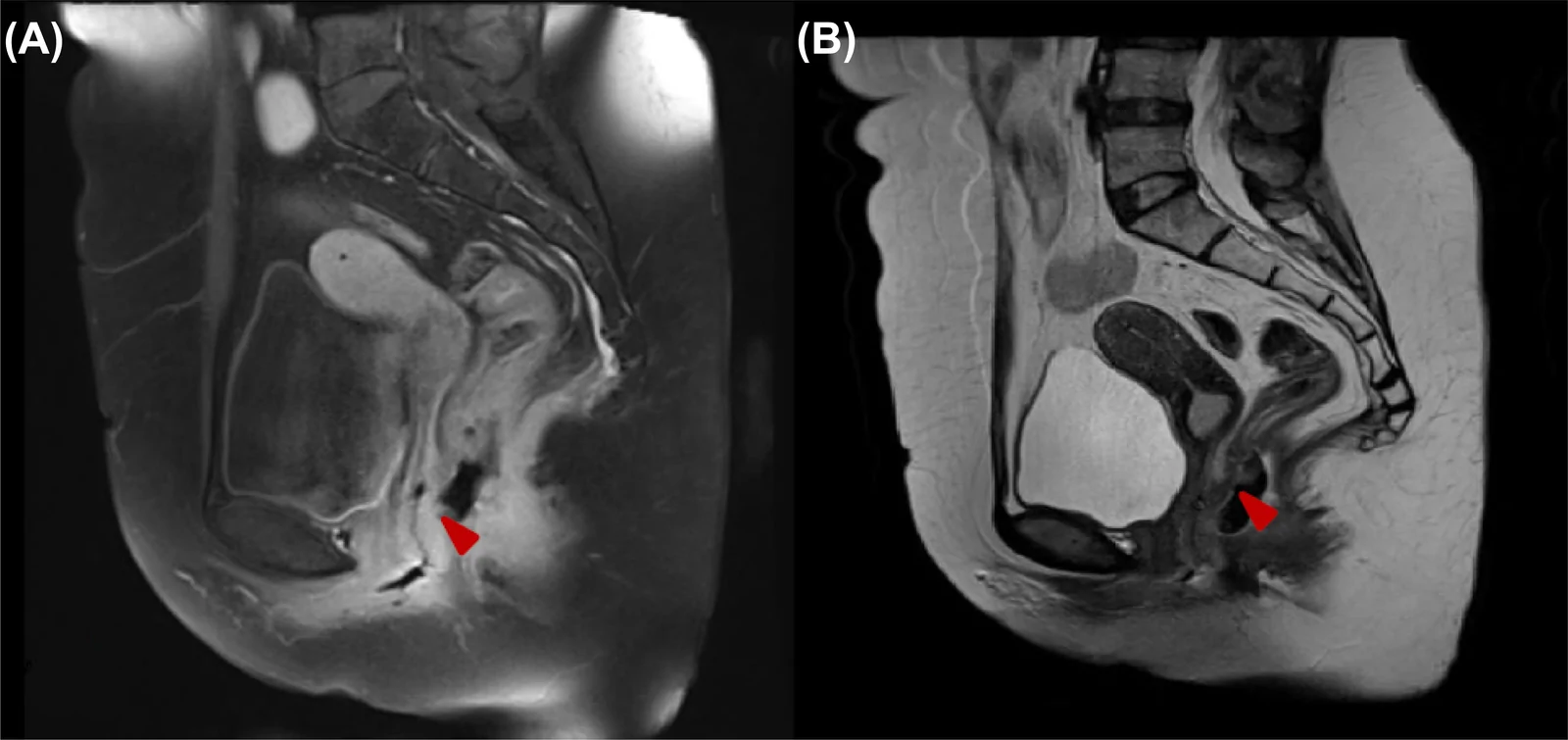
**A:** Sagittal T1 TSE post contrast with FS. **B:** Sagittal T2-weighted TSE magnetic resonance imaging of the pelvis presents a fistula tract that extends through the anterior rectal wall to the posterior of the uterus; however, it was not clear enough to confirm it (red arrow).

**Fig. (3):**
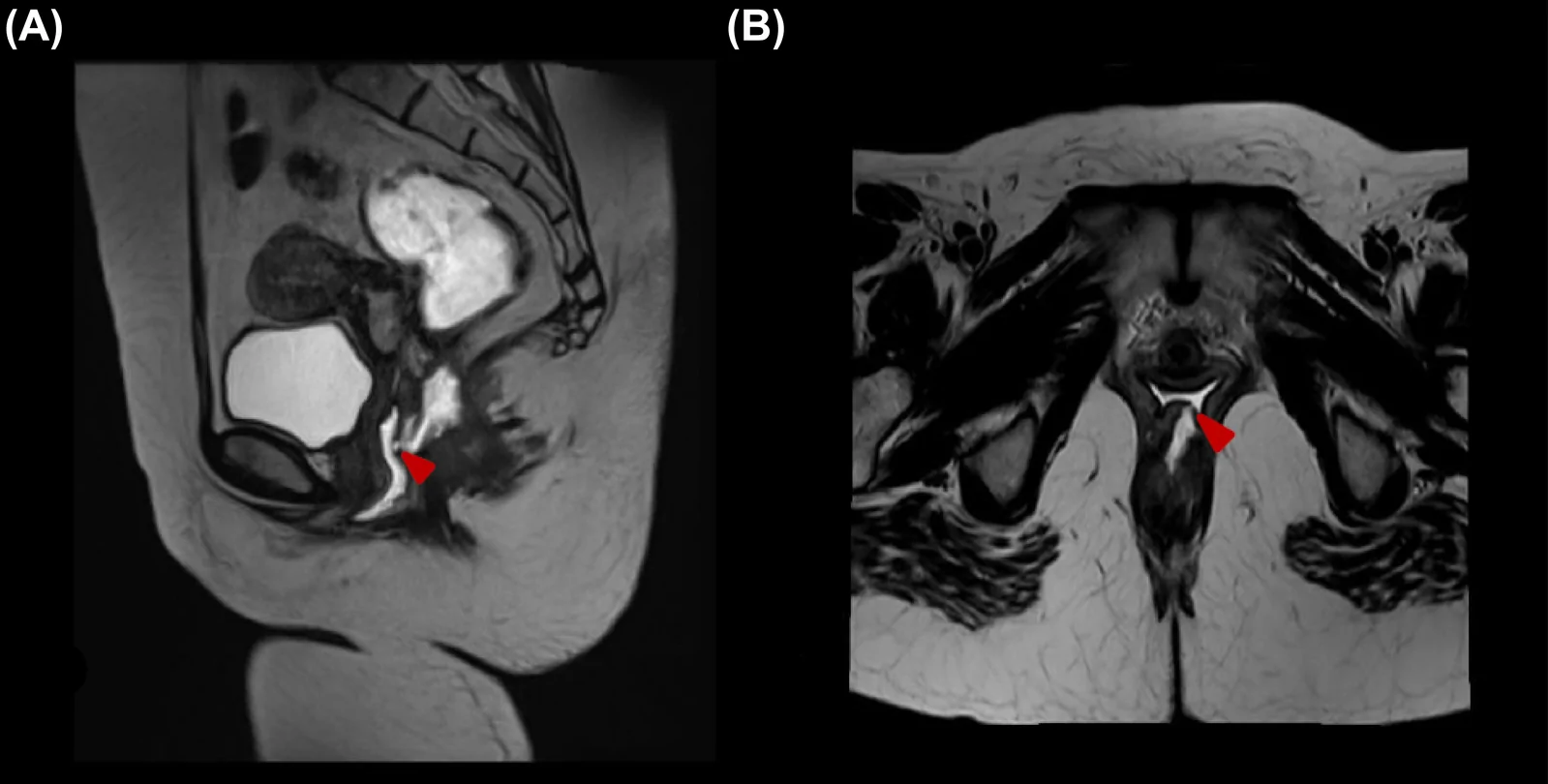
**A:** Sagittal T2-weighted TSE **B:** Axial T2-weighted TSE magnetic resonance imaging (MRI) after injection of ultrasound gel revealed a hyperintense fistulous tract extending between the anterior rectal wall and the posterior uterine wall.

**Table 1:** The MRI imaging sequences obtained before and after contrast injection.

-	**TR**	**TE**	**Slice Thickness**	**Flip Angle**	**Phase**	**FOV**	**GAP**	**NEX**
**Pre-contrast injection sequences**								
T1 TSE coronal	500	11	4	150	R-L	300	30	2
T1 TIRM coronal	2060	33	6	150	R-L	280	30	2
T2 TSE Sagittal	4550	77	5	150	A-P	254	30	2
T2 TSE axial FS	5010	70	5	150	A-P	310	30	2
T2 TSE axial	4350	70	5	150	A-P	310	30	2
**Post-contrast injection sequences**								
T1 TSE FS axial	701	9.1	5	150	A-P	299	60	2
T1 TSE FS coronal	701	9.1	4	150	R-L	313	60	2
T1 TSE Sagittal	713	9.4	4.5	150	A-P	276	60	2

**Abbreviations:** TIRM: turbo inversion recovery magnitude, TSE: Turbo spin echo, FS: Fat saturation

**Table 2:** The MRI imaging protocol after administration of ultrasound gel rectally.

-	** TR **	** TE **	** Slice Thickness **	** Flip Angle **	** Phase **	** FOV **	** GAP **	** NEX **
T2 TSE axial	5010	70	5	150	A-P	310	30	2
T1 TIRM coronal	2060	33	6	150	R-L	310	30	2
T2 TSE coronal	3540	70	5	150	R-L	310	10	2
T2 TSE sagittal	4550	77	4.5	150	A-P	254	30	2
T2 SPC RST ISO sagittal	1500	101	1	150	A-P	310	0	2

**Abbreviations**: SPC: Single-slab Pulsed Constant, RST: Reynolds stress tensor, ISO: isotropic

## Data Availability

The original contributions presented in this study are included in the article.

## LIST OF ABBREVIATIONS

RVF: Rectovaginal Fistulas
MRI: Magnetic Resonance Imaging
TSE: Turbo Spin Echo
FS: Fat Saturation

## STANDARDS OF REPORTING

CARE guidelines were followed.
